# Nutritional implications of feeding free‐living birds in public urban areas

**DOI:** 10.1111/jpn.13441

**Published:** 2020-09-19

**Authors:** Sara A. Burt, Cornelis J. Vos, Jan A. Buijs, Ronald J. Corbee

**Affiliations:** ^1^ Faculty of Veterinary Medicine Institute for Risk Assessment Sciences Utrecht University Utrecht The Netherlands; ^2^ Department of Companion Animal Health Faculty of Veterinary Medicine Utrecht University Utrecht The Netherlands; ^3^ Municipal Health Service Amsterdam The Netherlands

**Keywords:** bread, ducks, feeding, gulls, pigeons, rats

## Abstract

Supplementary feeding can affect populations of birds. It reduces energy spent on foraging and reduces the risk of starvation, but it also increases the risk of disease transmission and predation. Supplementary feeding may reduce species richness if some species are better able to exploit supplementary food resources than others. Feeding may also artificially inflate the carrying capacity of the ecosystem, leading to bird nuisance in the form of droppings and noise. The aim of this study was to characterise and quantify the risk factors and consequences of feeding free‐living birds in public areas in the western part of the city of Amsterdam. In seven study areas, the following data were collected: bird population size and species composition, feeding events, and the type and amount of supplementary food offered. Estimations were made of the nutritional energy provided and the number of birds that could be supported by the food offered. Members of the public who fed the birds were invited to complete a questionnaire on various aspects of feeding. Results showed that supplementary feeding attracts juvenile gulls and feral pigeons, which could in the long‐term affect biodiversity. Bread was the main category of supplementary food being offered (estimated to be 67% of the total amount of food). The majority of respondents fed birds so as not to waste bread and meal leftovers. In six of the seven areas studied, an overabundance of nutritional energy was calculated. We conclude that the current type and extent of supplementary feeding in the city of Amsterdam is nutritionally unbalanced and affects species diversity at a local level. The overabundance is undesirable for reasons of both animal health, because it can lead to malnutrition, and public health, because surplus food attracts rats and may also have a negative effect on water quality.

## 1 INTRODUCTION

In some public areas of Amsterdam, wild birds are fed all year round by local people. Feeding the birds is something many people do for enjoyment (Lawson et al., 2017; Warnken, Hodgkison, Wild, & Jones, 2004) or because they think the birds need the food, but reasons such as atonement for environmental damage have also been given (Howard & Jones, 2004). Several species of birds are attracted to the food on offer, but whether they benefit from the extra nutrients is debatable, since a large part of the food is anthropogenic. If so much food is put out that the birds cannot consume it all, it pollutes the watercourses (Hermsen, Maessen, van der Pouw Kraan, & Hendriks, 2011) and other animals, such as rodents, can be attracted by the surplus.

Not all species are attracted to anthropogenic food to the same extent (Plummer, Risely, Toms, & Siriwardena, 2019). Accessibility of food in urban areas is therefore probably the most critical factor in the regulation of bird populations (Galbraith, Beggs, Jones, & Stanley, 2015; Newton, 1998; Plummer et al., 2019). Positive effects of deliberate feeding for individual birds are as follows: reduced time needed for foraging, improved body condition and increased reproductive output (Chamberlain et al., 2009; Robb, McDonald, Chamberlain, & Bearshop, 2008). However, negative effects of exploiting anthropologic food are also possible: increased disease transmission (Fischer & Miller, 2015; Pennycott et al., 2007), dependence on the supplementary food resource (Lahti, Orell, Rytkönen, & Koivula, 1998), malnutrition (Ishigame, Baxter, & Lisle, 2006), and increased nitrogen and phosphate load in the water (Hermsen et al., 2011). This negative effect on water ecology will also affect bird health. Overall, feeding of wild birds may have both advantages and disadvantages for the bird population. Although the people feeding may experience pleasure from the activity, there can be public health risks attached, such as the transmission of zoonoses (Burt, Roring, & Heijne, 2018; Tsiodras, Kelesidis, Kelesidis, Bauchinger, & Falagas, 2008), which creates costs for the public.

Several studies on feeding of wild birds have focused on feeding in private gardens, but few have focused on urban situations (Amrhein, 2014; Clark, Whitney, MacKenzie, Koenen, & DeStefano, 2015; Davies, Fuller, Dallimer, Loram, & Gaston, 2012; Jones & Reynolds, 2008; Lepczyk, Mertig, & Liu, 2004). In private gardens, factors tending to increase feeding activity are the age of the householder, having a detached house and having a large family (Davies et al., 2012). However, research on the motivations of urban people in feeding birds is scarce (Clark et al., 2015; Jones & Reynolds, 2008).

We suspected that the amount of supplemental food being offered at some sites in Amsterdam could be detrimental to bird health and may have public health consequences. To investigate this, insight is needed in species richness, feeding events, and the amount of food being offered. We therefore aimed to characterize and quantify the risk factors and consequences of feeding free‐living birds as a first step. The specific aims of the present study were (a) to record the species and numbers of birds in seven study areas in the western part of Amsterdam, (b) to estimate the type and amount of supplementary food offered and its influence on the species present, (c) to compare the nutritional energy value in the food offered to the requirements of the birds and (d) to explore the motivations of people who put out food.

## 2 MATERIALS AND METHODS

The study took place from August to October 2016. The selection of study areas was based on recent reports of bird feeding made by citizens to the municipality, and at the request of the alderman responsible for animal welfare. The location of the seven study areas within the western part of the city of Amsterdam is shown in Appendix S1. The study areas were centred on a particular canal or square where feeding had previously been reported and stretched outwards up to and including the first block of buildings around it. The length of the study areas including a canal was determined by the road bridges across the canal. Every area was surveyed at least seven times. Each 90‐min survey consisted of a 15‐min bird census immediately followed by a 75‐min observation period to record any feeding events. Only one survey was made per day and location. In total, 54 surveys were carried out. The surveys were carried out on bicycle during daylight hours.

### 2.1 Population size and species composition

Each survey started with a 15‐min bird census, in which the number and species of birds present along the central transect were recorded. All birds present in the water or on the adjacent street, lawns, and on top of buildings were noted. The juveniles of *Larus argentatus* and *Larus fuscus* were grouped together because the difference between juveniles of the species was not apparent within the limited time available for counting during feeding events.

### 2.2 Food offered and numbers and species attracted

Immediately after the bird census, the study area was observed for 75 min to record any feeding events that occurred. For each feeding event, the type of food offered was registered, the amount of food was estimated and the species and numbers of birds attracted to the food were recorded. The amount of food was estimated by observation of the amount fed according to the following scale: a handful (approx. 50 g), half a bread bag (approx. 400 g), whole bread bag (approx. 800 g), plastic carrier bag (approx. 1,600 g) and big shopper (approx. 3,200 g). Participation ratios for each bird species were calculated by dividing the number of birds of a particular species that approached and/or consumed the food by the total number of birds of that species counted during the initial census.

### 2.3 Estimation of metabolizable energy provided by supplementary feeding

For each study area, the mean daily abundance of metabolizable energy (kJ ME) available from supplementary feed fed during the observation periods was calculated. For this, we first calculated the total amount of ME fed during our observations based on the energy value and the amount of food fed. We assumed a typical feeding period of 10 hr per day and extrapolated the amount of food during the 75 min of observation to a 10‐hr period. Because it was not practically possible to determine the type and energy value of all food offered, we used the energy value of bread (Kollias & Kollias, 2010) for leftovers. The energy value of bread was taken as 1,073 kJ ME/100 g, which is comparable to the energy value stated in a previous study (1,004 kJ ME/100 g, Orros & Fellowes, 2015). For bird species with significant participation ratios, we then calculated the total daily maintenance requirement (MER) of the birds counted in the initial census using published data on avian nutritional requirements and body mass (Klasing, 1998; Sales & Janssens, 2003). Finally, the mean daily surplus of kJ ME was calculated by comparing the total number of kJ ME in the available food with the total MER of the birds counted in the initial census. This difference was then compared to the MER of the brown rat (*Rattus norvegicus*) (Subcommittee on laboratory animal nutrition, 1995) to arrive at the number of rats that could, theoretically, be supported by the additional food. We cannot exclude that this food may have been eaten by birds that flew in late, or from other areas, or was consumed by the birds in excess of their requirements and stored as fat. This is the maximal potential food surplus available for rats.

The nutritional value of bread was also compared with the nutritional recommendations for avian foods for the species recorded in this study. If data for these species were not available, we compared data for a related species or, if that was also unavailable, data for the chicken (*Gallus gallus*).

### 2.4 Questionnaire on feeding habits

People who were observed feeding birds were invited to complete a short questionnaire to explore their motivations for feeding and to find out what the target species were and the type and amount of food they offered (Appendix S2).

### 2.5 Statistics

A correlation between the amount of food offered and number of birds attracted to the food was tested for using a curve estimation in SPSS with all models that contain the value zero, including linear, cubic, quadratic and logarithmic. Participation ratios between species were compared by ANOVA and Tukey post hoc test. The level of significance was set at *p* < .05.

## 3 RESULTS

### 3.1 Population size and species composition

**Figure 1:**
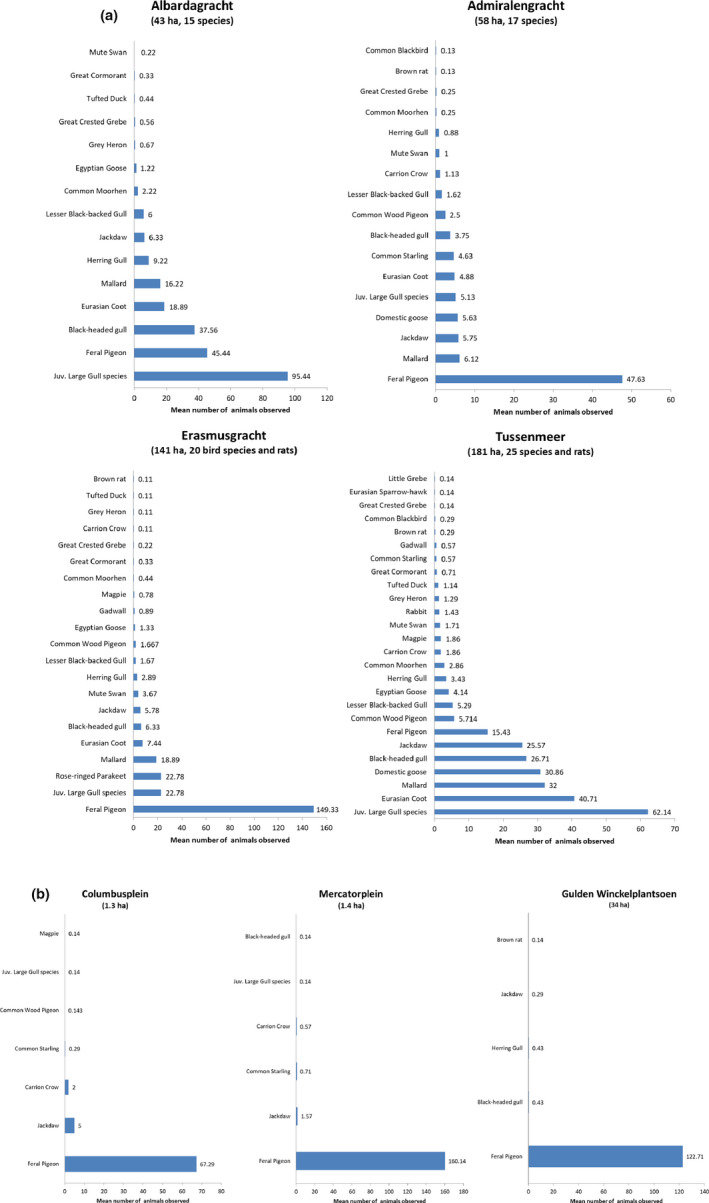
Species composition of (a) study areas containing a large body of water (b) study areas with no surface water. Numbers are means of all observations including feeding events [Colour figure can be viewed at wileyonlinelibrary.com]

The study areas containing a large watercourse showed a markedly higher species richness (14–24 species) compared to the areas without a watercourse (4–8 species) (Figure 1). The number of mammal species was at most two: brown rats (*Rattus norvegicus*) and rabbits (*Oryctolagus cuniculus*). Rats were recorded in three water‐rich areas and one ‘dry’ area, and rabbits only in one water‐rich area. These species were observed in the study areas irrespective of feeding events.

### 3.2 Food offered and numbers and species attracted

**Figure 2:**
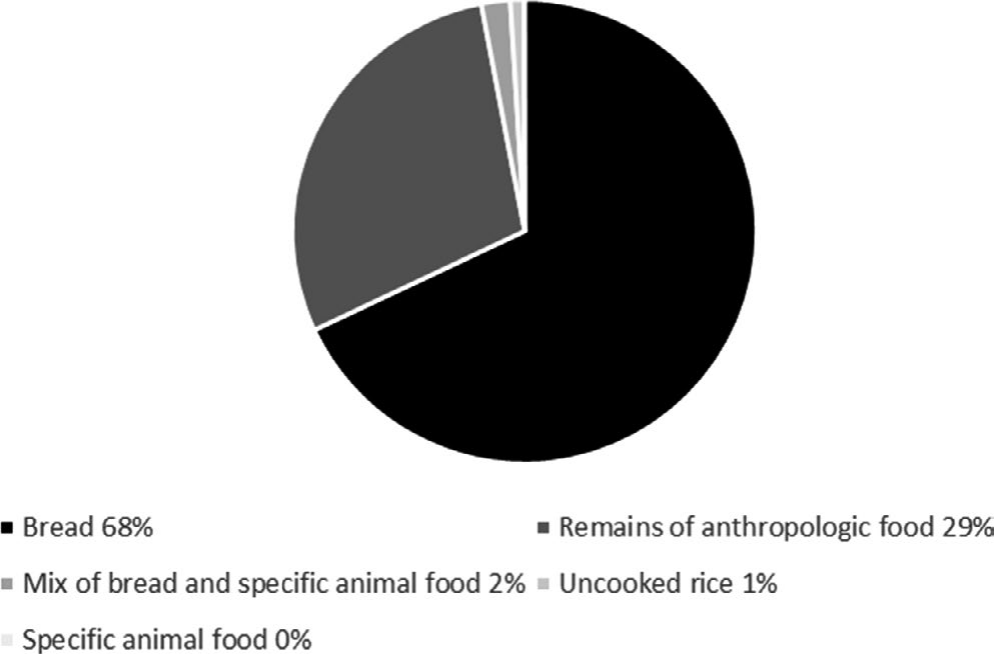
Types of food offered to the birds based on an estimate of weight. A total of 88 kg food was estimated to have been put out for all observation periods together

**Figure 3:**
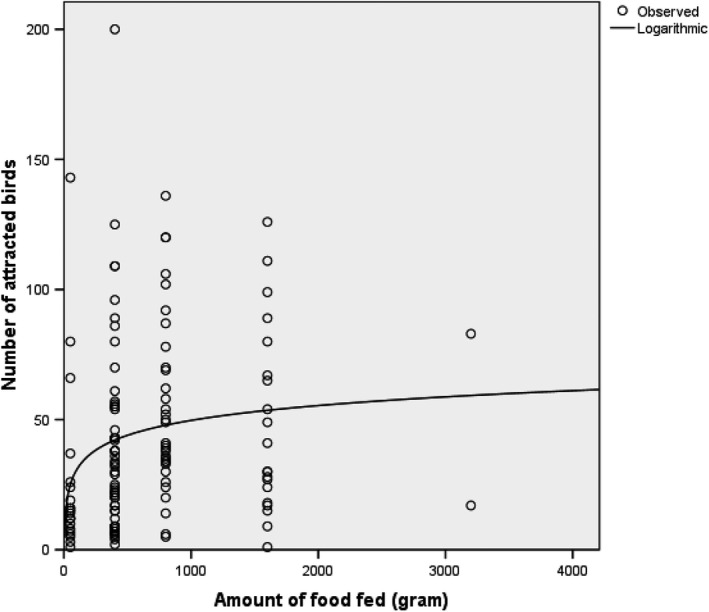
Correlation between the amount of food put out and the number of birds attracted to feed during the observation periods. The numbers of birds attracted to the food revealed that the numbers of birds attracted to a feeding event increased with increasing amount of food offered to around 50 and then levelled off, which fitted a logarithmic curve with best significance level (*p* = .004)

**Figure 4:**
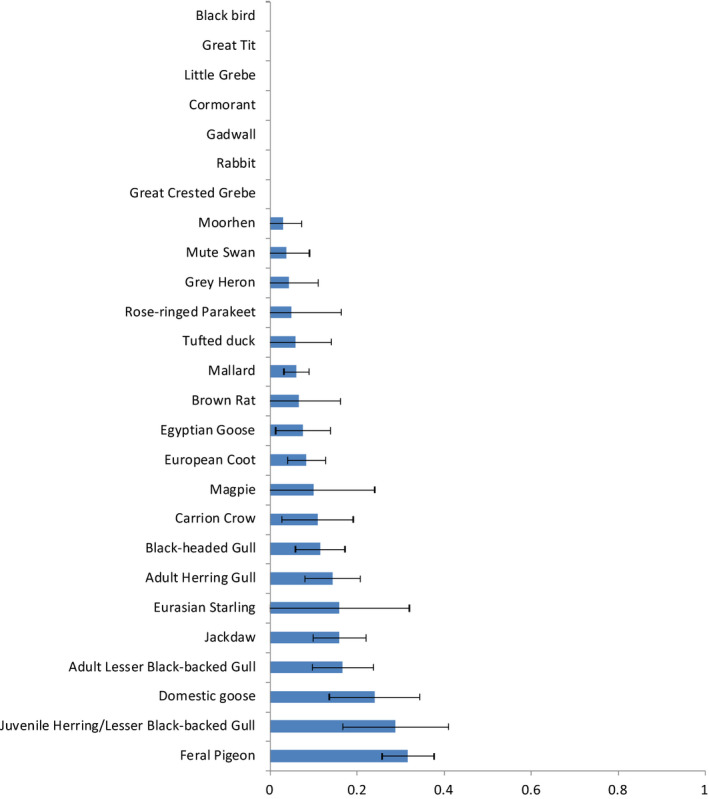
Feeding participation ratios for all species recorded (the mean proportions of birds present that were attracted to the food). Error bars indicate the 95% CI [Colour figure can be viewed at wileyonlinelibrary.com]

An estimated total of 88 kg food was offered (Figure 2) during a total of 110 feeding events. The mean number of feeding events per survey or study area was two. Bread was the most popular food, followed by leftovers from meals. Specific animal food was not recorded. The numbers of birds attracted to the food revealed that the numbers of birds attracted to a feeding event increased with increasing amount of food offered to around 50 and then levelled off, which fitted a logarithmic curve with best significance level (*p* = .004) compared to other curve types (linear, cubic and quadratic options in SPSS) that were able to contain the value zero for number of birds. (Figure 3). Inter‐species differences in feeding participation were apparent (Figure 4, Appendix S3). Ten bird species had participation ratios whose 95% confidence intervals did not include zero, and of these feral pigeons (*Columba livia domestica*), juvenile gulls and domestic geese were the top three. Eight species had participation ratios that were not significant, and seven species (6 bird, 1 mammal) did not participate in feeding at all; these were songbirds, some water birds and rabbits (Figure 4 and Appendix S3). In the water‐rich study areas, the species that were present in greatest numbers were also the species that had the highest participation ratios (Figures 1a and 4), that is juvenile gulls and feral pigeons.

### 3.3 Estimation of metabolizable energy provided by supplementary feeding

The ratio of white bread to wholemeal bread collected was approximately 1:1 in all study areas; therefore, the mean of the energy values of white and wholemeal (1,071 kJ/100 g) was used for calculations (Kollias & Kollias, 2010). The estimated mean daily energy available per study area during the observed feeding events was calculated to be 145 MJ ME. Subtracting the mean daily MER of the birds counted in the initial census (109 MJ ME) resulted in a mean energy surplus of 36 MJ ME per study area. In view of the fact that the daily maintenance requirement for a brown rat is approximately 236 kJ (Subcommittee on laboratory animal nutrition, 1995), the surplus of food would, on average, provide enough energy to maintain approximately 153 rats per study area.

The comparison of the nutritional value of bread with the published advice on the composition of avian food is presented in Appendix S4. Bread contains a sufficient amount of methionine, cysteine, magnesium and vitamin B1 for the target species. However, it is, on an energy basis, deficient in protein and fat content, calcium, phosphorus, several vitamins and some trace minerals. Bread is too rich in sodium and chloride to be suitable for birds.

### 3.4 Questionnaire on feeding habits

Out of a large number of people who were observed feeding during the surveys, 34 were willing to fill in the questionnaire (Appendix S5). Almost all respondents (97.1%) lived in the immediate neighbourhood. The top three targeted species in descending order were pigeons (35.3%), gulls (29.4%) and ducks (20.6%), but 35.3% of respondents did not target a particular species. Bread was stated to be the most frequently offered food (67.4%) followed by bread + leftovers (17.6%), leftovers (11.8%) and specific animal food (2.9%).

The majority (55.9%) of respondents said their reason for feeding the birds and animals was because they had leftover food. Almost a quarter (23.5%) did so because they thought the animals needed the food. Almost half (44.1%) of respondents said they fed the birds because they had learned from their parents that they should not waste food. About three quarters (73.5%) of respondents said that the amount of food being fed was determined by the amount of meal leftovers they had. About a quarter (26.5%) said they based the amount of food they fed on the number of birds present at the site.

Exactly half of respondents said they did not know whether the leftover food was good for the birds and animals, 44.1% thought that it was good for them, and a minority (5.9%) thought that it was not good for them. The majority (61.8%) said they would be prepared to feed less if asked to do so. Around a fifth (17.6%) of people said they would be prepared to feed more natural foodstuffs if asked to do so, but for a third of respondents (35.3%), it would depend on the costs involved. More than half (55.9%) of respondents could not name one of the species of birds they were feeding but 38.2% could name at least one. A large majority (70.6%) did not experience any nuisance from the animals they fed. Almost a third said they had experienced some nuisance from the animals being fed: brown rats and feral pigeons were most often named.

## 4 DISCUSSION

### 4.1 Population size and species composition

The study areas with a large body of surface water had markedly higher species richness compared to the dry study areas. This can be explained by the smaller surface area of the dry study areas and the lack of water to attract water birds.

The distance from a particular urban area to the edge of the city can be used as a measure for the degree of isolation for wildlife species, which generally leads to lower species richness (MacArthur & Wilson, 1967). For our data, species richness did not appear to be directly related to the distance to the edge of the city. This could be due to the relatively small sample size, or because the designated areas were relatively close to each other. The birds present in the different locations can therefore not be seen as separate populations.

### 4.2 Food offered and numbers and species attracted

Analysis of the types and amount of food offered revealed that the greatest part was human food, chiefly bread and meal leftovers, and that specific animal feed was not offered. A similar predominance of human leftover food was recorded in two studies in the United States and the Netherlands (Clark et al., 2015; Hermsen et al., 2011). In a study of food scattered for birds in US parking lots, bread, baked products and French fries were in most of the feedings (Clark et al., 2015) and of the 555 people observed leaving food behind, 36 (6.5%) dumped the food and left without waiting to see whether birds ate the food (Clark et al., 2015). In a study of sources of water pollution in three towns in the Netherlands, people arriving in cars were also seen to empty bags of bread and immediately drive away (Hermsen et al., 2011).

A plot of the estimated amount of food offered against the number of birds attracted (Figure 3) showed that increasing the amount of food did not lead to a directly proportional increase in the numbers of birds attracted. The number of birds levelled off at about 50; above this, a further increase in the amount of food did not result in a corresponding rise in the numbers of birds attracted. We hypothesize that this may be due to spatial reasons—more than 50 birds may not easily access the food at the same time if it is placed on the ground. In any case, if the food is to be consumed by the birds, it would be best if the amount of food on offer at any one time was limited. Offering more than this may tend to result in surplus.

The participation perc for feral pigeons was 0.31, which is similar to the findings of a previous study (0.36, Sol & Senar, 1995). The species that were present in greatest numbers during the initial census were also the species that had the higher participation ratios, namely gulls and feral pigeons (Figures 2a and 4). We hypothesize that the populations of these species benefit most from supplementary feeding. This may have caused a flock effect, resulting in other species present in the surrounding area not daring to visit the study areas. Another explanation for this difference in species richness could be access to other (more natural) food sources, other plant species or other water bodies.

### 4.3 Estimation of metabolizable energy provided by supplementary feeding

The mean estimated daily surplus of nutritional energy was 36 MJ ME per study area. The accuracy of this estimate depends on how representative the 75‐min observation periods are for whole 10‐hr periods, and whether the leftovers will be targeted by other bird species after the primary flock has gone. Rodent mammals such as the brown rat are known to consume edible garbage (van Adrichem, Buijs, Goedhart, & Verboom, 2013), and we calculated that 153 rats per study area (1,071 rats for all seven study areas together) could be sustained by this overabundance of food. Although the results of the questionnaire indicate that the majority of respondents intend to feed the birds, it is clear that at least some of the food they scatter is potentially sustaining rats rather than birds.

Although rats had a small, non‐significant participation ratio (Appendix S3), our feeding observations were made during daylight hours, and since brown rats are more active at night (Bonmati‐Carrion, Baño‐Otalora, Madrid, & Rol, 2017), we expect that they would have a much higher participation ratio at night. A surplus of bread and leftovers at the end of the day may therefore sustain rodent populations. This would be undesirable due to the risk of disease transmission to animals and man. Further, brown rats are also known to predate on the chicks and eggs of waterfowl (Armitage, 2017). Although this is normal predatory behaviour, when there is abundance of rats, this could further contribute to the skewing of the natural balance of species in these urban bird populations.

Based on our comparison of the nutritional value of bread with published recommendations for avian feed, bread is an unsuitable food for birds (Appendix S4). Presumably few or no birds would be eating a diet of bread alone, so the nutritional consequences in the study areas may not be serious. However, no research has yet been carried out on the proportions of bread in the diet. We propose that this should be a topic for further research, as other researchers have called for (Jones & Reynolds, 2008). Such data would be useful to determine how much urgency should be attributed to reducing overabundant feeding of birds.

### 4.4 Questionnaire on feeding habits

The order in which the various categories of food were said to be offered agree largely with our recordings of food found in the study areas (Figure 2). People feed the birds very close to home and mostly in order to dispose of bread and leftovers from meals. They perceive it as a way of ‘not wasting food’. A majority would be amenable to feeding less or feeding more natural food (depending on costs) if requested to do so. This concurs with a study in the USA, where 75% of 141 people interviewed whilst feeding birds said they would stop leaving food for the gulls (Clark et al., 2015). Interestingly, using up leftover food was not among the reasons cited by respondents in a survey of bird feeders in Australia (Howard & Jones, 2004). There, ‘pleasure’ was the most frequent reason and ‘environmental atonement’ was also frequently mentioned.

For the respondents to our questionnaire, the top three species they targeted were, in descending order: pigeons, gulls and ducks (Appendix S5). The participation ratios of pigeons (0.32) and juvenile gulls (0.29) are, indeed, greater than other species (Appendix S3). However, the participation ratios of mallard (*Anas platyrhynchos*) and tufted duck (*Aythya fuligula*) in this study were very low (both 0.06), which may be due to the ducks being more active at night. There is some evidence that these species are mostly active at night, although this is not always the case for mallards (Bengtsson et al., 2014; Dirksen, Spaans, van der Winden, & van den Berg, 1998; Sauter, Korner, Fiedler, & Jenni, 2012).

Interestingly, although pigeons were the number one targeted species for feeding, pigeons and brown rats were most often mentioned as being a source of nuisance in the neighbourhood (Appendix S5). Here lies a conflict: people take pleasure in feeding the pigeons (and other birds), but also experience nuisance from their large numbers and from rats attracted by the overabundance of food. It is usually very difficult to persuade people to stop feeding by prohibition and/or educational methods; social marketing methods focused on the local community have been suggested as a more successful method (Clark et al., 2015). We suggest using behavioural insights may be an appropriate route (Thaler & Sunstein, 2008). The use of behavioural insights involves adapting information streams and/or the environment so that the public is more likely to exhibit desirable behaviours (Quigley, 2013).

### 4.5 Further research

In order to investigate whether local birds suffer any nutritional deficiencies due to the overabundance of human food, it would be necessary to estimate what proportion of their daily diet is derived from bread and leftovers. This could be estimated by attaching a camera to a bird to record what it eats in the course of a day. To find out what effect feeding has on species richness, data on the species richness at popular feeding locations could be compared to areas with a similar ecological layout but no supplemental feeding.

Although the solutions to urban bird problems can be relatively simple (Huig, Buijs, & Kleyheeg, 2016), persuading people to change their behaviour can be difficult. Previous research has shown that prohibitive and/or educational signing most likely will not be sufficient to significantly reduce feeding activities (Clark et al., 2015). It would be useful to investigate the efficacy of behavioural insights in persuading local inhabitants to put out less bread and leftovers for the birds.

## 5 CONCLUSION

In conclusion, we can say that supplemental feeding on the present scale, although it may be of certain advantage to birds on an individual level, may also be harmful to them if they experience nutritional imbalances. Populations of feral pigeons and gulls appear to benefit most from supplementary feeding. The number of birds attracted by feeding levels off at a certain number, even if food for more birds is available, which can lead to surplus food remaining as litter, or subsequent feeding by other birds or mammals. Based on the energy value of the food on offer and the energy needs of the birds, at the majority of sites a surplus of food is available, which may benefit certain bird species but could also help to sustain rodent populations. The nutritional value of the food offered is not a good match to the needs of wild bird species and could lead to malnutrition if eaten to the exclusion of natural food. People scatter food close to home and mostly in order to dispose of bread and leftovers from meals, targeting pigeons, gulls and ducks. There is a conflict between the convenience and pleasure people derive from feeding leftover food to the birds and the nuisance they experience from pigeons and rats attracted to it.

## CONFLICT OF INTEREST

The authors declare no conflict of interest.

## ANIMAL WELFARE STATEMENT

The ethical policies of the journal have been adhered to, the appropriate ethical approval has been received, and that the research meets EU standards for the protection and use of animals for scientific purposes and/or feed legislation. The study was approved by the institute of animal welfare as required under Dutch legislation. Because this study was not regarded as an animal experiment, no further ethical approval was required.

## Supporting information

Supplementary Material
