# Supplementary material for: Nutritional implications of feeding free‐living birds in public urban areas
Source: J Anim Physiol Anim Nutr (Berl). 2020 Sep 19;105(2):385–93. doi: 10.1111/jpn.13441 (PMC7984256; doi:10.1111/jpn.13441)
Supplement: Supplementary file 1 — Supplementary Material [file JPN-105-385-s001.docx]

Appendix S1 Map of the city of Amsterdam


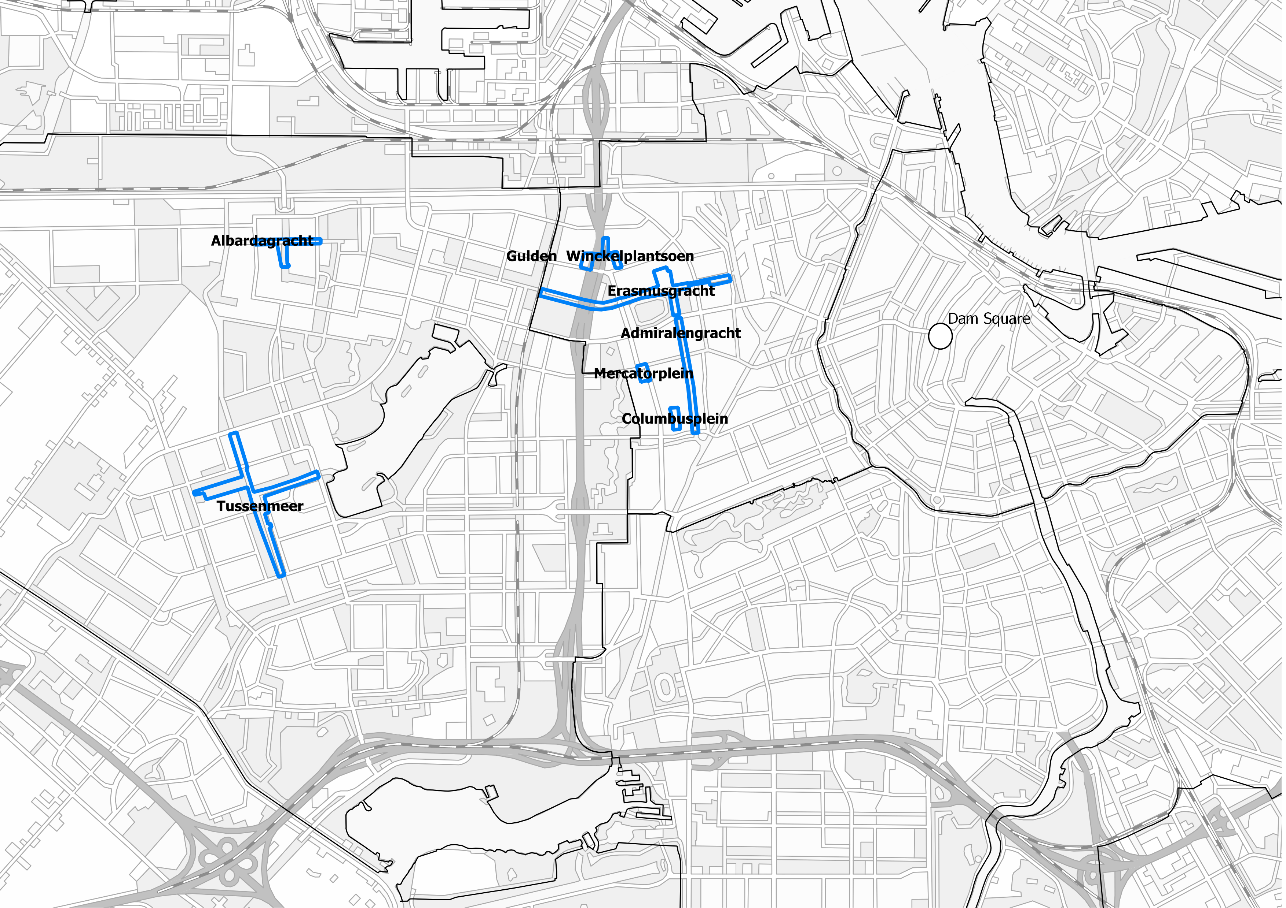


Map of the central and western parts of the city of Amsterdam showing the seven study areas marked in blue. Dam Square is the centre of the city. All research areas are located in the West of Amsterdam. Of the total of seven locations, four; Admiralengracht (52° 22' 4.379" N 4° 51' 26.698" E), Albardagracht (52° 22' 48.601" N 4° 48' 6.239" E), Erasmusgracht (52° 22' 32.239" N 4° 50' 50.777" E) and Tussen Meer (52° 21' 28.08" N 4° 47' 54.73" E) can best be described as canal including waterside. The other three locations; Columbusplein (52° 21' 55.292" N 4° 51' 19.476" E), Gulden Winckelplantsoen (52° 22' 44.245" N 4° 50' 49.348" E) and Mercatorplein (52° 22' 10.88" N 4° 51' 3.834" E) can best be described as street or square without a water body.

Appendix S2 Questionnaire

How many times do you feed?

Which animals do you feed?

Do you know the species by names?

What kind of foods do you feed?

Why do you feed?

What determines the quantities which you feed?

Why did you start feeding?

Do you think anthropologic food is good for animals?

Have you ever experienced nuisance of animals?

If you are asked to feed the animals less, would you?

If you are asked to feed the animals more natural food, would you?

Do you live in this neighbourhood?

What is your highest level of education?

Appendix S3 Feeding participation ratios for species recorded during the observation periods

|  | Ratio | 95% Confidence interval of the Difference |  |  |
| --- | --- | --- | --- | --- |
|  |  |  |  |  |
|  |  | Lower | Length of bar | Upper |
| Feral Pigeon* | 0.32 | 0.26 | 0.06 | 0.38 |
| Juvenile Herring/Lesser Black-backed Gull^#^ | 0.29 | 0.17 | 0.12 | 0.41 |
| Domestic goose | 0.24 | 0.14 | 0.10 | 0.34 |
| Adult Lesser Black-backed Gull | 0.17 | 0.10 | 0.07 | 0.24 |
| Eurasian Jackdaw | 0.16 | 0.10 | 0.06 | 0.22 |
| Eurasian Starling | 0.16 | -0.00 | 0.16 | 0.32 |
| Adult Herring Gull | 0.14 | 0.08 | 0.06 | 0.21 |
| Black-headed Gull | 0.11 | 0.06 | 0.06 | 0.17 |
| Carrion Crow | 0.11 | 0.03 | 0.08 | 0.19 |
| Eurasian Magpie | 0.10 | -0.04 | 0.14 | 0.24 |
| Eurasian Coot | 0.08 | 0.04 | 0.04 | 0.13 |
| Egyptian Goose | 0.08 | 0.01 | 0.06 | 0.14 |
| Brown Rat | 0.07 | -0.03 | 0.09 | 0.16 |
| Mallard | 0.06 | 0.03 | 0.03 | 0.09 |
| Tufted duck | 0.06 | -0.02 | 0.08 | 0.14 |
| Rose-ringed Parakeet | 0.05 | -0.06 | 0.11 | 0.16 |
| Grey Heron | 0.04 | -0.02 | 0.06 | 0.11 |
| Mute Swan | 0.04 | -0.02 | 0.06 | 0.09 |
| Common Moorhen | 0.03 | -0.01 | 0.04 | 0.07 |
| Great Crested Grebe | 0 |  |  |  |
| Rabbit | 0 |  |  |  |
| Gadwall | 0 |  |  |  |
| Great Cormorant | 0 |  |  |  |
| Little Grebe | 0 |  |  |  |
| Great Tit | 0 |  |  |  |
| Eurasian Blackbird | 0 |  |  |  |

Inter-species differences in participation ratios in feeding occasions for birds and mammals in the city of Amsterdam. Participation ratios represent the mean proportion of a population that actually got attracted to feeding occasions. Lower and upper bar of the 95% confidence interval of the difference in ratios were also given. ***** significantly more compared to Grey Heron, Eurasian Jackdaw, Lesser Black-backed Gull, Mute Swan, Black-headed Gull, Tufted Duck, Eurasian Coot, Egyptian Goose, Common Moorhen, Mallard, Herring Gull, Carrion Crow. ^#^ significantly more compared to Grey Heron, Eurasian Coot, Egyptian Goose, Common Moorhen, Mallard, and Carrion Crow.

Appendix S4 Comparison of the nutritional value of bread with published recommendations for avian feed

Bread contains on average 2565kcal ME per kg and has the following nutrient composition on an as fed basis:

Macronutrients: *crude protein* 9.6%, *ether extract* 3.1%, *linoleic acid* 0.002%, *crude fiber* 1.0%, **nitrogen-free** **extract** 48.2%,

Minerals: *Calcium* 0.09%, *Phosphorus* 0.17%, Magnesium 500mg/kg, *Potassium* 0.19%, **Sodium** 0.52%, **Chloride** 0.79%,

Trace elements: *Copper* 1.7mg/kg, *Iodine* 0.14mg/kg, *Iron* 14mg/kg, *Manganese* 9mg/kg, *Selenium* 0.028mg/kg, *Zinc* 10mg/kg,

Vitamins: *vitamin A* 15IU/kg, *vitamin D* 0.0IU/kg, vitamin E 5.22IU/kg, *vitamin K* 0.055mg/kg, vitamin B1 4.5mg/kg, vitamin B2 2.5mg/kg, Niacin 45.5mg/kg, *vitamin B6* 1.5mg/kg, *Pantothenic acid* 4.5mg/kg, *Biotin* 0.00074mg/kg, Folic Acid 0.805mg/kg, *Choline* 205.5mg/kg.

Nutrients in *Italic* are deficient for all investigated species, Nutrients underscored are deficient for some investigated species, Nutrients in **bold** are excessive for all investigated species. Data obtained of Vaessen & Ooik (1990), Vaessen et al. (1991), National Research Council (1994), Jansen & Nijboer (2003), Staggs et al. (2004), Abou Khashaba et al. (2009), Kollias & Kollias (2010), Voedsel en Waren Autoriteit (2010), United States Department of Agriculture (2015), and Bath & Rayman (2016).

References

Abou Khashaba, H.A, Sayed, M.A.M., Mariey, Y.A., Ibrahem, M.A. (2009). Nutritional and management studies on the pigeon: Estimate of Metabolizable energy requirements. Egyptian Poultry Science 29, 481- 501.

Bath, S., Rayman, M. (2016). Food Fact Sheet Iodine. The British Dietetic Association (BDA). Retrieved from <https://www.bda.uk.com/foodfacts/Iodine.pdf>

Jansen, W.L., Nijboer, J. (2003). *Zoo Animal Nutrition Tables and Guidelines*. EZNC: Amsterdam.

Kollias, G.V., and Kollias, H.W. (2010). Feeding Passerine and Psittacine Birds. In M.S. Hand, C.D. Tatcher, R.L. Remillard, P. Roudebush, B.J. Novotny (Eds.), Small animal clinical nutrition, (pp 1255-1269). Mark Morris Institute: Topeka, Kansas.

National Research Council. (1994). *Nutrient Requirements of Poultry*. The National Academies Press: Washington, D.C.

Staggs, C.G., Sealey, W.M., McCabe, B.J., Teague, A.M., Mock, D.M. (2004). Determination of the biotin content of select foods using accurate and sensitive HPLC/avidin binding. Journal of Food Compostion and Analysis 17, 767–776, *doi*: 10.1016/j.jfca.2003.09.015

United States Department of Agriculture, 2015. *USDA National Nutrient Database for Standard Reference*. Retrieved from <https://ndb.nal.usda.gov/ndb/>

Vaessen, H.A.M.G., Ooik, A. 1990. Bread - Iron and selenium content. RIVM Report 388801005. Retrieved from <https://www.rivm.nl/publicaties/brood-gehalte-aan-ijzer-en-selenium>

Vaessen, H.A.M.G., Ooik, A., Tolsma, K., Loon, J.W. 1991. Bread - Copper, Manganese and Zinc content. RIVM Report 389101001. Retrieved from <https://www.rivm.nl/publicaties/brood-gehalte-aan-koper-mangaan-en-zink>

Voedsel en Waren Autoriteit. 2010. Monitoring van het gehalte aan keukenzout in diverse levensmiddelen. Factsheet Project ZD10 1614. Retrieved from <https://www.voedingscentrum.nl/Assets/Uploads/Documents/Voedingscentrum/Professionals/VWA-monitoring-zout-in-levensmiddelen-2010.pdf>

Appendix S5 Responses to the questionnaire that was given to people observed putting out food

| **Factor** | **Category** | **Survey** Count | **Respondents**  % |
| --- | --- | --- | --- |
| **Frequency** | Several times a day Daily Several times a week Weekly Occasionally | 2 7 13 2 10 | 5.9 20.6 38.2  5.9 29.4 |
| **Target species*** | Ducks Gulls Pigeons Geese Mute Swans Eurasian Jackdaws  Grey Heron No specific species | 7 10 12 6 3 2 1 12 | 20.6  29.4  35.3  17.6  8.8  5.9  2.9  35.3 |
| **Bird knowledge** | Recognizes species Does not recognize species | 13 19 | 40.6 59.4 |
| **Food type** | Bread Bread and leftovers Leftovers Specific animal food | 23 6 4 1 | 67.4 17.6 11.8 2.9 |
| **Reason behind feeding** | Think animals need it Because I have leftovers Companionship Outing with (grand)children | 8  19  5  2 | 23.5  55.9  14.7  5.9 |
| **Determination of quantity** | Quantity of leftovers Population size  Amount of money | 25  9  0 | 73.5  26.5  0 |
| **Reason behind starting to feed** | Learned from parents  Saw animals starving  Did not have anything else to do  Don’t know why | 15  6  7  6 | 44.1  17.6  20.6  17.6 |
| **Knowledge of animal nutrition** | Leftovers are not good  Leftovers are good Don’t know | 2  15  17 | 5.9  44.1  50 |
| **Nuisance** | Yes, (mainly Brown Rat and Feral Pigeon  No | 10  24 | 29.4  70.6 |
| **Motivation to start feeding less** | Yes  No  I don’t know | 21  11  2 | 61.8  32.4  5.9 |
| **Motivation to start feeding more natural** | Yes  No  Depends on price  Depends on convenience | 6  12  12  1 | 17.6  35.3  35.3  2.9 |
| **Residency** | Same neighborhood as feeding  Other location | 33  1 | 97.1  2.9 |
| **Highest Qualification**** | MBO lever or lower  Higher education level | 11  2 | 84.6  15.4 |

*NB Percentage adds up to more than 100 since respondents could select multiple options.

** N is 13 for this particular question.

Only residents that were actually feeding were being asked to participate.
